# Empyema Necessitatis: A Rare Complication of Empyema

**DOI:** 10.7759/cureus.48973

**Published:** 2023-11-17

**Authors:** Gabriella Gerlach, Rachel E Garrity, Guillermo Izquierdo-Pretel, Efren Buitrago

**Affiliations:** 1 Internal Medicine, Florida International University, Herbert Wertheim College of Medicine, Miami, USA; 2 Cardiovascular Thoracic Surgery, Jackson Memorial Hospital, Miami, USA

**Keywords:** empyema, empiric antibiotics, video-assisted thoracoscopic surgery (vats), pulmonary decortication, thoracic empyema, empyema necessitatis

## Abstract

Empyema necessitatis (EN) is an exceedingly rare complication of empyema. EN refers to the expansion and progression of an empyema beyond the thoracic cavity toward the skin wall. Herein, we present the case of a man with EN and detail his clinical course. A 42-year-old male with a prior history of substance use presented to the emergency department with three weeks of fever, cough, and progressively worsening pain overlying the left anterior chest wall. An empiric antibiotic regimen of cefepime, metronidazole, and vancomycin was initiated. Chest X-ray, ultrasound, and chest CT demonstrated a large region of loculation suspicious for a loculated empyema. On day 4 of admission, he underwent a video-assisted thoracoscopy followed by a left minithoracotomy, which confirmed the diagnosis of EN. The patient was discharged on hospital day 16 with marked clinical improvement and monitored for a year via an outpatient clinic. Symptoms did not recur, and there was complete resolution of EN. More predominant in the pre-antibiotic era with the progression of uncontrolled infections, EN is less commonly seen today. As such, EN requires a high degree of clinical suspicion for timely detection and management. Our case illustrates the importance of early intervention with antibiotics and surgical drainage.

## Introduction

Empyema necessitatis (EN) is an exceedingly rare complication of empyema. It is defined as the expansion of a thoracic empyema through nearby soft tissues toward the chest wall, sometimes dissecting through the skin. Most commonly, EN will present as a persistent mass in the thoracic wall, noticeable on physical exam [1]. The raised mass can be erythematous, pruritic, and painful leading clinical suspicion to include superficial skin infections [1]. More chronic cases can present with fistulas communicating with the pleural space, as EN has gone either undetected or untreated [2].

First identified by Hippocrates, EN was more predominant in the pre-antibiotic era, as untreated empyema would progress and expand chronically without drainage [3]. Since the advent of highly effective antibiotics, cases of EN have greatly diminished, but continue to persist especially in developing nations. *Mycobacterium tuberculosis* is regarded as the most common infectious etiology for EN, and thus, areas endemic for this pathogen continue to see EN cases [1]. Even in developed nations, clinical suspicion for EN should remain high, especially in medically underserved regions. Common comorbidities that may predispose patients to EN include chronic lung diseases and bronchopulmonary infections, diabetes mellitus, immunosuppression, malnutrition, and thoracic trauma [1]. EN classically has a prolonged latency period with symptoms that are often nonspecific, making timely diagnosis a challenge if EN is not considered early in the disease course [4]. We present the case of a man with EN and detail his clinical course including history at admission, labs, imaging, antibiotic regimen, surgical treatment, and follow-up period.

## Case presentation

**Figure 1:**
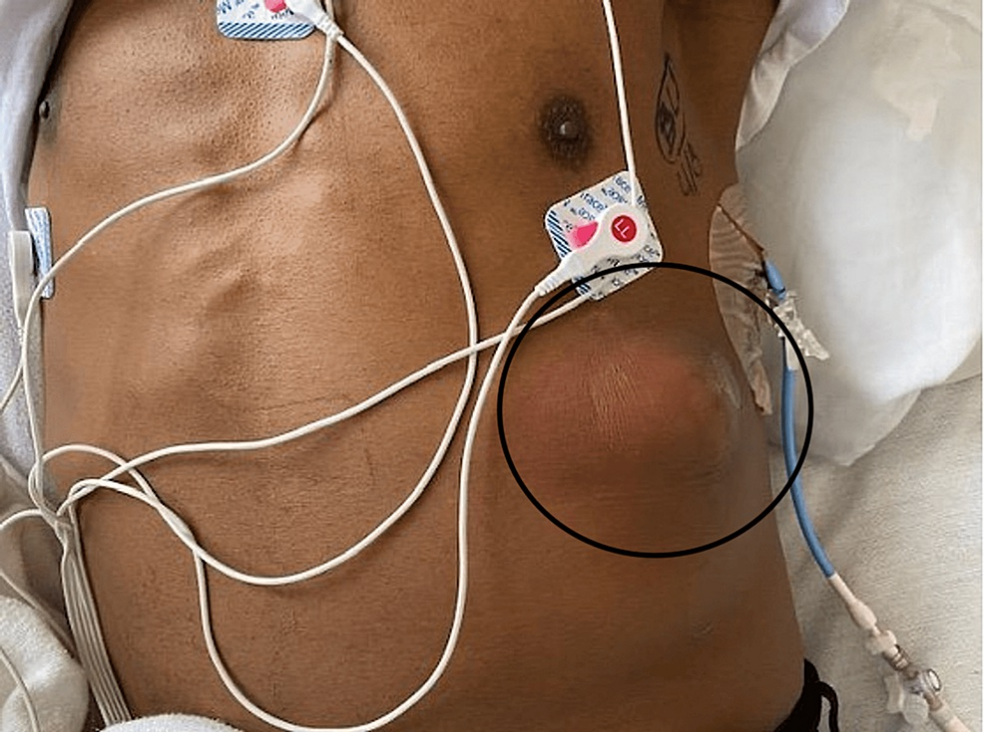
Fluctuant mass on the left anterior costal area.

A 42-year-old male presented to the emergency department with three weeks of fever, cough, and progressively worsening pain overlying the left anterior chest wall. The patient had noticed a mass-like lesion, and accompanying skin changes appear over the past week which motivated him to come to the emergency room. He had a past medical history of substance use including alcohol and crack cocaine, but denied intake for a month prior to this admission. On physical exam, his temperature by mouth was 38.2°C, pulse 115, respiration 19 breaths per minute, blood pressure 123/81 mmHg, and BMI 20.7. The patient was well-developed though found to be pale and thin. A painful, raised, fluctuant mass of 5 cm in diameter was noted on the left anterior costal area, as shown in Figure 1. Lungs were clear to auscultation bilaterally. Bilateral cervical lymph nodes as well as a left-sided supraclavicular lymph node and a left-sided axillary lymph node were small, but mildly enlarged and firm. Cardiac, respiratory, abdominal, and neurological physical exam findings were within normal limits. In the emergency room, a pigtail catheter was inserted into the left side of the chest, which immediately drained a yellowish, purulent effusion.

Laboratory exam on admission revealed a marked anemia with a hemoglobin of 6.6 g/dL and a hematocrit of 22%. The patient was transfused with one unit of packed red blood cells, which increased his hemoglobin to 7.9 g/dL. Complete blood count suggested the possibility of a bacterial infection with an elevated white blood cell count of 14.3 x 10^3^ mcL, elevated C-reactive protein (CRP) of 23.3 mg/dL, elevated erythrocyte sedimentation rate (ESR) of 141 mm/h, and elevated procalcitonin of 0.246 ng/mL. Albumin of 2.6 g/dL was concerning for malnutrition. Pleural fluid analysis was highly suggestive of empyema with a gross appearance of turbid, purulent fluid, elevated lactate dehydrogenase (LDH) of 3,926 U/L, pleural protein of 4.6 g/dL with a pleural-to-serum protein ratio of 0.54, and elevated white blood cell count of 9,426 mcL with 79% neutrophils. Cytologic evaluation of this effusion was negative for malignancy, and surprisingly, cultures were negative for bacterial or fungal growth, including acid-fast bacilli. The patient was admitted for further evaluation and treatment of the left anterior chest wall mass and a complex pleural effusion of undetermined etiology. On admission, he was started empirically on a 14-day course of 2 g of intravenous (IV) cefepime every eight hours, 500 mg of IV metronidazole every eight hours, and IV vancomycin. Vancomycin was initially started at 1 g every 12 hours and gradually increased to therapeutic levels at 1.5 g every eight hours.

**Figure 2:**
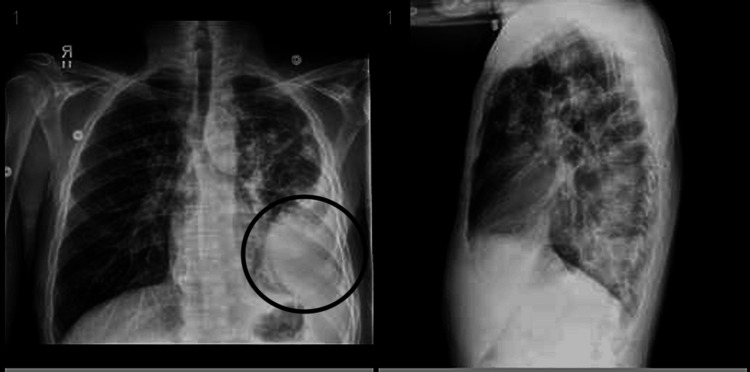
Chest X-ray on admission, showing a large opaque density projecting over the left lower hemithorax.

**Figure 3:**
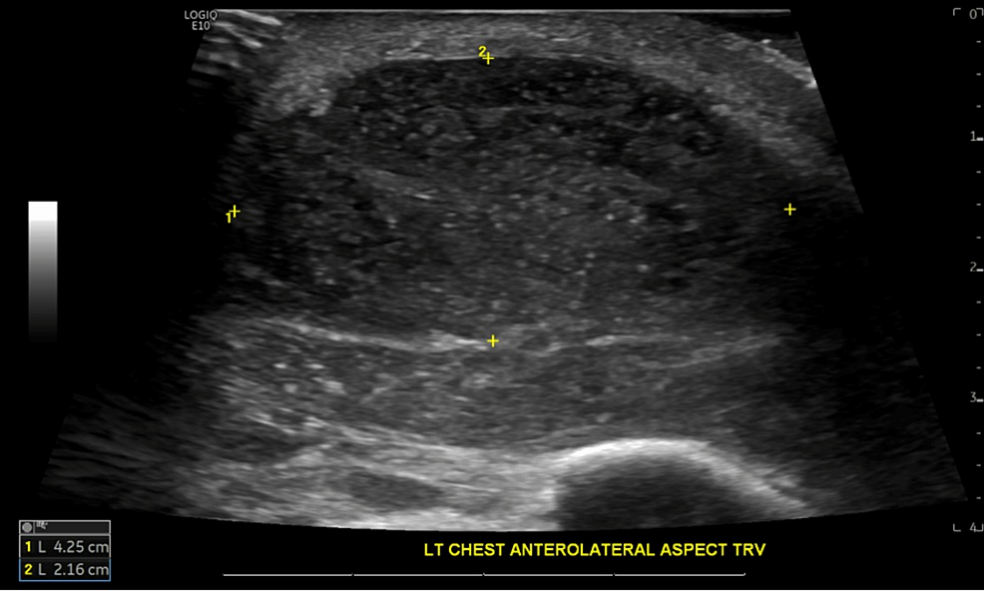
Chest ultrasound on day 1 of admission, showing a heterogeneous oval-shaped mass within the anterolateral aspect of the left chest wall.

**Figure 4:**
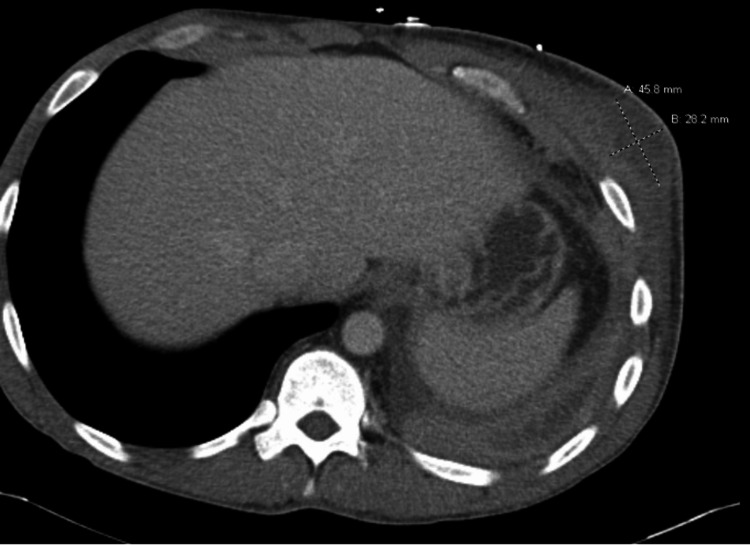
Chest CT on day 2 of admission, suspicious for loculated empyema.

Chest X-ray on day 1 showed a large density projecting over the left lower hemithorax measuring 2.3 x 7.8 cm, as shown in Figure 2. Ultrasound of the chest on day 1 of admission revealed a heterogeneous oval mass within the anterolateral aspect of the left chest wall measuring 4.3 x 3.8 x 2.2 cm, approximately 3 mm beneath the skin, as shown in Figure 3. CT chest with contrast on day 2 of admission demonstrated an area of large loculation with central hypodensity containing a mixture of simple to more complex fluid with surrounding rim of enhancement in the left lower lobe, suspicious for a loculated empyema. CT also demonstrated a left lower anterior chest wall hypoenhancing ovoid structure measuring approximately 4.6 x 2.8 cm with associated fat stranding of the surrounding tissue and an additional, more inferior lesion measuring 2.8 x 1.5 cm, as shown in Figure 4.

 

 

On day 4 of admission, the patient underwent thoracoscopy via video-assisted thoracoscopic surgery (VATS) followed by a left minithoracotomy by the surgical team. He required extensive lysis of adhesions, decortication, left pleural and lung biopsies, evacuation of the empyema, and irrigation and debridement of the chest wall abscess. The diagnosis of EN was confirmed, and a wound vacuum dressing was placed over the surgical site. Pathologic analysis showed fragments of fibroadipose tissue and lung parenchyma with fat necrosis, as well as exuberant acute and chronic inflammation with associated fibrosis. There was no evidence of malignancy or other lymphoproliferative disorder. His wound vacuum was successfully removed on day 16 of admission, and he was discharged home the same day. He was seen in the outpatient clinic on day 28. He was then followed for 11 months in the outpatient clinic with no return of clinical symptoms and reported complete resolution of disease course.

## Discussion

EN is a rare complication of empyema that develops as an infection progresses through the thoracic cavity and subcutaneous tissues toward the chest wall. EN can frequently be avoided if pulmonary infection is identified and treated early, but can progress to empyema and then ultimately EN if left undiagnosed or improperly treated [5]. EN typically presents as a large, palpable subcutaneous mass that causes the skin of the chest wall to protrude, overlying the initial empyema [3]. The mass is often fluctuant with associated erythema and pain. Accompanying changes to the consistency of adjacent lymph nodes are also common, as seen in this case. Other less specific features of presentation may include cough, fever, fatigue, and dyspnea. Although EN can present as a mass on the chest wall with hardened regional lymph nodes, a differential that includes cellulitis, necrotizing fasciitis, malignancy, and lymphoproliferative disorders must be considered.

The initial workup for cases of EN should include anteroposterior and lateral chest X-rays, but CT of the chest with contrast is essential for more accurate diagnosis [3]. Ultrasound of the chest is a quick modality that can be used at the bedside to further assist in the diagnosis. Pleural fluid analysis is required for the categorization of the parapneumonic effusion as an empyema and can begin to screen for underlying malignancy with the evaluation of cytology results [6]. Most importantly, pleural fluid analysis provides an opportunity to analyze cell count and culture the causative organism guiding a targeted effective antibiotic regimen. With EN, an exudative effusion is expected, given the infectious etiology is most often bacterial. According to Light's criteria, pleural fluid would have at least two of the following: a pleural fluid protein-to-serum protein ratio greater than 0.5, a pleural fluid-to-serum LDH ratio greater than 0.6, or a pleural fluid LDH greater than the upper limit of normal for serum LDH by a factor of 2/3 [6]. However, negative culture results do not necessarily exclude infectious causes of EN as organisms may be difficult to isolate. In fact, it is not uncommon for no causative organism to be identified in EN [7]. An infectious disease consultation should also be obtained. 

The most common causative organisms of EN today are *M. tuberculosis* and *Actinomyces israelii*, while other less common causative agents include methicillin-resistant *Staphylococcus aureus* (MRSA) and fungi [7]. In cases of suspected EN, empiric therapy is usually given until final culture results are available [7]. Our patient received IV vancomycin, cefepime, and metronidazole for 14 days. In the case of bacterial EN, the duration of treatment is highly dependent on the causative pathogen and can range from 10 days up to 18 months. In addition to targeting the causative pathogen, the duration of antibiotic treatment is also influenced by the extent of the lesion and the response to treatment [2]. The antibiotic choice is also highly specific for the causative pathogen, if blood or pleural fluid cultures yield a positive result. In our case, multiple blood and collected effusion cultures were unfortunately negative. Upon further review, the patient was started empirically on antibiotics at the time of admission, which likely interfered with the ability of the cultures to grow the underlying organisms. However, pathology findings showed extensive inflammatory changes without evidence of malignant cells, suggesting EN with a non-granulomatous bacterial etiology. Our patient likely had some degree of immunocompromise as he was noted to be malnourished on admission and had a history of long-term substance use, which likely predisposed him to the complicated pneumonia and EN.

Cases of EN that are not detected early typically require surgery for full resolution of the infection and retention of pulmonary function [3]. It follows that options for surgical management will differ based on individual patient characteristics and past medical history. Options for surgical management include simple tube drainage, open drainage, and partial decortication of the thoracic cavity. Simple tube drainage is accomplished via chest tube placement into the empyema. This is the least invasive method to evacuate the empyema and should be attempted prior to other surgical methods in all patients. Open drainage is preferred for older patients with chronic disease and is completed by inserting a large chest tube through separated ribs and irrigating with saline. Akgül et al. described pleural irrigation with saline for mechanical debridement of the cavity in these patients, as the empyema can be dense and difficult to drain by chest tube alone. Decortication is a surgical procedure done to remove fibrous, infected, or neoplastic tissue attached to the lung, pleural space, chest wall, or diaphragm. It can be used in younger patients to "obliterate the cavity" after persistence of the expansion for at least seven days [3]. With treatment including early administration of antibiotics and surgical intervention as needed, there have been no reported deaths from EN in the modern era [8].

## Conclusions

EN is a rare complication of empyema that can be seen in a variety of immunocompromised patients. The first steps in proper management include investigation for the source of infection and defining the extent of disease. This can be done by obtaining imaging via chest X-ray and chest CT, and analysis of laboratory studies, and most importantly pleural fluid analysis. Once the etiology is determined, medical management with antibiotic is the standard of care but may not be potent enough to cure the disease if EN was not diagnosed early. Successful treatment of more progressive cases of EN include both medical and surgical therapy. Our case identifies the importance of early identification, surgical treatment, and a team approach in the case of EN.
